# Role of T Cells in Malnutrition and Obesity

**DOI:** 10.3389/fimmu.2014.00379

**Published:** 2014-08-11

**Authors:** Valerie A. Gerriets, Nancie J. MacIver

**Affiliations:** ^1^Division of Pediatric Endocrinology and Diabetes, Duke University Medical Center, Durham, NC, USA

**Keywords:** obesity, inflammation, T cells, malnutrition, leptin

## Abstract

Nutritional status is critically important for immune cell function. While obesity is characterized by inflammation that promotes metabolic syndrome including cardiovascular disease and insulin resistance, malnutrition can result in immune cell defects and increased risk of mortality from infectious diseases. T cells play an important role in the immune adaptation to both obesity and malnutrition. T cells in obesity have been shown to have an early and critical role in inducing inflammation, accompanying the accumulation of inflammatory macrophages in obese adipose tissue, which are known to promote insulin resistance. How T cells are recruited to adipose tissue and activated in obesity is a topic of considerable interest. Conversely, T cell number is decreased in malnourished individuals, and T cells in the setting of malnutrition have decreased effector function and proliferative capacity. The adipokine leptin, which is secreted in proportion to adipocyte mass, may have a key role in mediating adipocyte-T cell interactions in both obesity and malnutrition, and has been shown to promote effector T cell function and metabolism while inhibiting regulatory T cell proliferation. Additionally, key molecular signals are involved in T cell metabolic adaptation during nutrient stress; among them, the metabolic regulator AMP kinase and the mammalian target of rapamycin have critical roles in regulating T cell number, function, and metabolism. In summary, understanding how T cell number and function are altered in obesity and malnutrition will lead to better understanding of and treatment for diseases where nutritional status determines clinical outcome.

## Nutritional Status Alters T Cell Immunity

Both obesity and malnutrition are major health problems around the globe. The World Health Organization has listed both obesity/overweight and childhood malnutrition/underweight on its top 10 causes of global mortality and disease. In developed countries, there has been an emphasis on the deleterious health effects of obesity. Obesity is associated with life-threatening co-morbidities including cardiovascular disease and type 2 diabetes that shorten lifespan. However, we must remember that for the majority of the world population, malnutrition from chronic food deprivation is the more common nutritional problem. Both obesity and malnutrition are associated with changes in immune cell number and function that alter immunity and have consequences for infection and inflammation. In this review, we will discuss the effects of nutrition on T cell distribution and function and examine the role of T cells in altered immunity in both malnutrition and obesity.

## Obesity and Inflammation

Obesity is a growing epidemic in developed countries. For example, over 30% of Americans are currently classified as overweight or obese (1). Unfortunately, the obesity epidemic is accompanied by a myriad of associated health risks. Exacerbating the problem is the absence of safe and effective treatments to reverse obesity. Lifestyle modifications such as diet and exercise are oftentimes ineffective, and at present, bariatric surgery is the most effective treatment for obesity but can be associated with post-surgical complications and side effects.

Obesity is associated with life-threatening co-morbidities such as insulin resistance leading to type 2 diabetes mellitus, as part of the metabolic syndrome (2). Indeed, the prevalence of both obesity and diabetes has increased in parallel during the last 20 years, and is highly associated (3). From these trends, the United States Centers for Disease Control (CDC) predicts that 1 in 3 American adults will have diabetes by the year 2050 (4). As part of the metabolic syndrome, obese individuals are also at risk for hyperlipidemia and hypertension leading to increased cardiovascular and renal disease. Additionally, obesity increases the risk of multiple forms of autoimmunity (5–7), including multiple sclerosis (MS), thyroid autoimmunity, and type 1 diabetes. Obesity is further associated with an increased risk of certain forms of cancer, including esophageal, breast, endometrial, colorectal, kidney, pancreatic, gallbladder, and thyroid cancer (8, 9). And finally, obese individuals have increased susceptibility to infections due to impaired host defense (10). In fact, obese individuals were more susceptible to the pandemic H1N1 influenza outbreak of 2009 (11).

**Figure 1:**
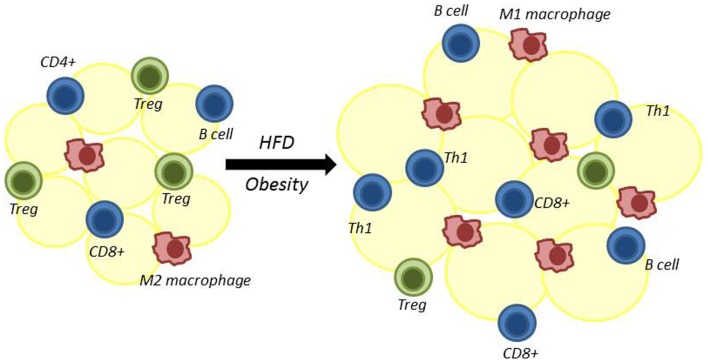
**Adipose tissue is remodeled following high-fat diet-induced obesity**. Multiple immune cells reside in adipose tissue. High-fat diet-induced obesity results in decreased regulatory T cells (Treg) and alternatively activated M2 macrophages. At the same time, there is an increase in adipose-resident inflammatory macrophages (classically activated M1 macrophages), CD4+ Th1 cells, CD8+ T cells, and B cells. The role of CD4+ Th2 and Th17 cells in adipose tissue in obesity is less certain.

Obesity is associated with both systemic inflammation and an influx of pro-inflammatory immune cells into visceral adipose tissue (VAT) (12). These VAT-localized immune cells secrete inflammatory cytokines, which have been shown to promote insulin resistance (12, 13). It is now clear that T lymphocytes (T cells), in particular, have a critical role in the early stages of this inflammatory process. Both regulatory and inflammatory T cells are found in VAT and influence the recruitment and function of other inflammatory cells into VAT, thereby contributing to changes in insulin sensitivity in obesity (Figure 1). For these reasons, it is critically important to determine how obesity and increased adiposity alter T cell distribution and function to drive inflammation.

### Early studies on obesity-related inflammation

In the 1990s, several observations were made that first linked obesity with inflammation. A key early study by Hotamisligil et al. demonstrated that expression of the pro-inflammatory cytokine tumor necrosis factor alpha (TNF-α) was increased in the adipose tissue of obese animals (14). Moreover, loss of TNF-α in obesity resulted in improved insulin sensitivity and glucose homeostasis (14–16). Not long after these initial findings with TNF-α, other pro-inflammatory cytokines were implicated. In a prospective study in 2001, both C-reactive protein (CRP) and IL-6 were found to be predictive of the development of type 2 diabetes in obese patients (17).

Soon after, macrophages were identified as an inflammatory cell of interest in obesity-associated inflammation. In 2003, it was discovered that many macrophage-specific genes were upregulated in both genetic and diet-induced forms of obesity in animals (18, 19). Around the same time, macrophages were found to accumulate in adipose tissue in obese animals (19). A few years later, macrophages in obese adipose tissue were ascribed an inflammatory phenotype that differs from the phenotype of macrophages in lean adipose tissue (20, 21).

These early studies have led to an understanding of multiple immune cells, chemokines, and cytokines contributing to the network of inflammation leading to insulin resistance and metabolic syndrome in obesity. Since these early studies, multiple pro-inflammatory cells, in addition to macrophages, have been ascribed a role in either promoting inflammation (lymphocytes, mast cells, neutrophils, and NK cells) or regulating inflammation (regulatory T cells) in obesity. Additionally, adipocytes themselves can secrete pro-inflammatory cytokines, many of which overlap with cytokines produced by macrophages, including TNF-α and IL-6, as well as hormones that influence inflammation, such as the pro-inflammatory hormone leptin and the anti-inflammatory hormone adiponectin.

An important question remained, how do inflammatory cytokines and signals promote insulin resistance? Elevated inflammatory signals were found to induce both c-Jun N-terminal kinase (JNK) signaling pathways and activation of the inhibitor of nuclear factor kappa-B (NF-κB) kinase subunit beta (IKKβ), which goes on to promote NF-κB activation. Both JNK and IKKβ/NF-κB signaling pathways were found to decrease insulin action of adipocytes and hepatocytes (22, 23). Multiple studies have now shown that blocking inflammatory cells or cytokine signaling in adipose tissue prevents development of inflammation and subsequent insulin resistance in obesity.

### Macrophages in obesity-related inflammation

Macrophages are present in both lean and obese VAT and, in fact, make up 40–60% of VAT immune cells in obesity. The role of the macrophage in VAT and promoting insulin resistance in obesity is well studied and has been extensively reviewed (24, 25). Interestingly, there is an overlapping biology and function of VAT-localized macrophages and adipocytes, and these two cell types produce many hormones and cytokines in common, including inflammatory cytokines implicated in obesity-associated inflammation and insulin resistance (26). These inflammatory cytokines have paracrine effects on other adipose-localized cells and may be secreted into circulation to exert endocrine effects systemically.

Macrophages are polarized toward either an M1 or M2 phenotype. Macrophages in lean tissue are typically M2 macrophages, also known as “alternatively activated macrophages,” which secrete anti-inflammatory cytokines, including IL-10, IL-1 receptor agonist, and arginase-1. Obesity-associated VAT macrophages are largely M1 macrophages, or “classically activated macrophages” that secrete large amounts of inflammatory cytokines including TNF-α, IL-6, IL-12, IL-1β, and monocyte chemotactic protein 1 (MCP-1) (24). These M1 macrophages in obese VAT are either derived from macrophages already present in adipose tissue, which have changed their phenotype in response to obesity, or they are recruited into adipose tissue from circulation. Obesity-associated effects on VAT include the release of chemokines, such MCP-1. MCP-1 binds to the chemokine (C–C motif) receptor 2 (CCR2) on macrophages, and has been described to recruit macrophages into adipose tissue in obesity (27). Many studies have demonstrated the role for classically activated M1 macrophages in promoting insulin resistance, and numerous mouse models have confirmed that disabling the macrophage inflammatory response pathway in obesity is protective against the development of insulin resistance (25, 28).

In addition to macrophages, other innate immune cells appear to participate in regulating inflammation and insulin resistance in obesity. Both pro-inflammatory neutrophils and mast cells are activated in obesity, and mice lacking these innate inflammatory cells are protected against insulin resistance (29). Conversely, eosinophil number is decreased in the VAT of obese mice, and eosinophil-deficient mice have increased inflammation and insulin resistance (29). While innate cells are clearly important to regulate inflammation in obesity, we will not focus on the role of the innate cells in obesity-associated inflammation and insulin resistance here, but rather highlight the role of the lymphocyte.

### Lymphocytes in obesity-related inflammation

In 2009, Nature Medicine published a series of three papers that altogether established a critical role for both inflammatory and regulatory T cells in mediating adipose tissue inflammation and altering insulin sensitivity in obesity (30–32). Together, these studies demonstrated that obesity was associated with T cell changes in VAT that included decreased regulatory T cells (Treg) and Th2 cells and increased inflammatory Th1 and CD8+ T cells. Soon after, other studies confirmed a role for T cells in adipose tissue in promoting systemic inflammation in obesity (33). These reports established a potentially early and critical role for inflammatory lymphocytes (CD8+ T cells, Th1 helper cells, and B cells) and regulatory lymphocytes (Th2 helper cells and Treg) in obesity-associated inflammation leading to insulin resistance. Thus began a new era of investigation into the role of lymphocytes in obesity-associated inflammation. It soon became clear that both the type and the proportion of lymphocytes and lymphocyte subsets change in VAT in obesity. We will discuss findings regarding lymphocyte subsets that either promote or down regulate obesity-induced inflammation here.

### Pro-inflammatory lymphocytes

#### CD4+ Th1 cells

VAT from high-fat diet-induced obese mice has higher levels of both CD4+ and CD8+ T cells, as compared to lean controls. T cells from obese VAT produce high levels of the Th1 cytokine interferon gamma (IFN-γ) when stimulated *in vitro* (34). IFN-γ secreted from adipose tissue induces the polarization of macrophages toward the M1 phenotype. IFN-γ also increases the production of other inflammatory cytokines, including TNF-α, from adipose tissue cultured *in vitro*. Obese animals lacking IFN-γ expression produced less adipose TNF-α and MCP-1, had decreased inflammatory cell accumulation in adipose tissue, and had improved insulin sensitivity compared to animals with normal IFN-γ expression (34, 35). Most recently, Khan et al. examined T cell receptor beta (TCRβ) deficient mice, which are protected against obesity-induced macrophage infiltration and insulin resistance, and found that adoptive transfer of Th1 cells into high-fat diet-fed TCRβ-/- mice led to increased muscle and adipose tissue inflammation as well as increased insulin resistance (36).

The T cell-specific T-box transcription factor, T-bet, which is known to induce Th1 development and promote transactivation of the IFN-γ gene, has also been implicated in obesity-associated inflammation. T-bet knockout mice have decreased energy expenditure and increased visceral adiposity compared to wildtype littermates, yet have better insulin sensitivity on both normal chow and high-fat diet (37). While the T-bet knockout mice were expected to have increased insulin sensitivity, the finding that they were more obese, with increased visceral adiposity, was surprising, as obesity and insulin resistance are typically co-associated. Improved insulin sensitivity is perhaps due to decreased expression of Th1 cytokines and increased expression of Th2 cytokines and the Th2 transcription factor GATA binding protein 3 (GATA3) in the T-bet knockout animal (38). Moreover, adoptive transfer of T-bet-deficient CD4+ T cells, but not wildtype CD4+ T cells, into Rag2-/- mice, which lack both B and T lymphocytes, modestly improves insulin sensitivity (37). As Th1 cells typically produce both T-bet transcription factor and IFN-γ cytokine, their role in insulin sensitivity was examined in IFN-γ knockout mice. Loss of T-bet on an IFN-γ knockout mouse conferred no additional improvement to insulin sensitivity (39).

#### CD4+ Th17 cells

The role of Th17 cells in VAT in obesity is less clear. In 2009, Winer et al. described an association between obesity and IL-17 production in mice (40). IL-17 expression has also been found to be high in obese human beings (41), and peripheral blood samples from human beings with type 2 diabetes mellitus have increased numbers of circulating Th17 cells (42, 43). Obesity can exacerbate autoimmune diseases that have a strong Th17-dependent mechanism, including experimental autoimmune encephalomyelitis (EAE) in mice, MS in human beings, and colitis (44). IL-17 has been found to be produced by T cells in adipose tissue, and IL-17 knockout mice are overweight and have increased obesity following high-fat diet compared to littermate controls, but show improved insulin sensitivity (45). However, IL-17 can be produced by both Th17 cells and gamma delta (γδ) T cells and appears to be secreted from γδ T cells in VAT (45). One group has even reported IL-17 production from neutrophils in obesity (46). Therefore, the role of Th17 cells in inflammation and insulin resistance in obesity remains uncertain.

#### CD8+ T cells

Obesity is associated with increased CD8+ T cells in adipose tissue (47). In 2009, Nishimura et al. demonstrated that CD8+ T cells preceded macrophages into VAT in obesity (30). Moreover, depletion of CD8+ T cells in diet-induced obesity resulted in decreased accumulation of macrophages into obese VAT as well as improved insulin sensitivity. Conversely, adoptive transfer of CD8+ T cells into CD8-deficient mice increased infiltration of macrophages into VAT as well as expression of the inflammatory cytokines IL-6 and TNF-α, along with development of insulin resistance following high-fat diet (30). These findings suggest a critical role for CD8+ T cells in the development of inflammation and insulin resistance in obesity. More recently, Jiang et al. confirmed VAT accumulation of CD8+ T cells in obesity and examined the mechanism of CD8+ T cell accumulation in adipose tissue (48). The authors found that VAT CD8+ T cells are activated *in vitro* by Th1 cytokine IFN-γ. Moreover, CD8+ T cells from VAT in obese mice expressed high levels of the integrin CD11a, which was important for infiltration of CD8+ T cells into adipose tissue in obesity (48).

#### B cells

In addition to increased CD4+ and CD8+ T cells, B cells also accumulate in VAT in diet-induced obesity (49). Diet-induced obese mice that lacked B cells, following treatment with B cell depleting CD20 antibody, were protected against insulin resistance, despite weight gain on a high-fat diet (50). Treatment of these B cell-deficient mice with IgG from wildtype obese mice resulted in restoration of insulin resistance (50). These findings, therefore, establish a role for both B cells and IgG antibodies in driving insulin resistance in obesity. In recent work from a separate group, cytokine production was compared in obesity from B cell null mice and wildtype controls. Obese B cell null mice had decreased inflammatory cytokines, including IL-6 and IFN-γ, increased anti-inflammatory IL-10 levels, and protection against insulin resistance compared to obese wildtype mice (51). Absence of B cells in obesity was also associated with an increased number of regulatory T cells (Treg) in VAT, as compared to obese wildtype mice. Additionally, human B cells from type 2 diabetics, but not from non-diabetic controls, were able to activate T cells *in vitro* (51). These results suggest a possible mechanism in which B cells precede T cells, and possibly macrophages, into VAT and regulate T cell differentiation, inflammation, and activation in obesity.

### Lymphocytes with an anti-inflammatory role in obesity

Regulatory T cells (Treg) have been found to have a key role in the regulation of inflammation and the development of insulin resistance in obesity. Obese human beings have decreased circulating Treg cells (52), and genetic mouse models of obesity and diet-induced obese mice show decreased Treg numbers in VAT (31). When Treg cells were depleted acutely, investigators observed increased transcription of inflammatory genes in VAT along with increased insulin levels and decreased insulin receptor signaling (31). This suggests that Treg play an important role to suppress obesity-related inflammation. Indeed, expansion of the Treg compartment in high-fat diet-fed mice was associated with increased levels of IL-10, statistically significant lower blood glucose levels, and trends toward lower insulin resistance and glucose tolerance (31).

In 2012, Cipolletta et al. described peroxisome proliferator-activated receptor gamma (PPAR-γ) expression as a critical player in Treg accumulation in VAT and in insulin sensitivity (53). PPAR-γ expression was increased in VAT Treg as compared to peripheral lymphoid Treg cells, and establishment of VAT Tregs from naïve CD4+ T cells depended on both PPAR-γ and Foxp3 expression. Indeed, conditional knockout of PPAR-γ in Treg resulted in decreased Treg number in VAT but did not affect Treg number in peripheral lymphoid tissue (53). PPAR-γ is well known to influence adipocyte differentiation and is considered anti-inflammatory. Members of the class of drugs called thiazolidinediones are PPAR agonists, and have been used in the treatment of type 2 diabetes mellitus for many years, although their use over the past five years has been limited due to cardiovascular side effects and outcome. The authors of this study found that PPAR-γ expression was required for action of the thiazolidinedione drug pioglitazone to restore insulin sensitivity in obesity, as pioglitazone enhanced the accumulation of VAT Treg and improved insulin sensitivity in obese mice (53). These findings suggest a novel mechanism by which PPAR agonists can promote insulin sensitivity through the regulation of T cell differentiation and function, as opposed to modulation of PPAR expression in adipocytes. Moreover, these findings highlight Treg as an interesting potential drug target of obesity-related inflammation and type 2 diabetes mellitus.

More recently, Han et al. described a key role for insulin in regulating Treg function (54). The authors found that insulin receptors are expressed on Treg, and that insulin signaling directly influenced Treg function by decreasing IL-10 production via activation of the AKT/mammalian target of rapamycin (mTOR) signaling pathway (54). Insulin was also able to reduce Treg suppression of TNF-α production by macrophages. When Treg were isolated from obese or lean VAT in mice, Treg from obese VAT produced less IL-10 and more IFN-γ than Treg from lean animals (54). These findings suggest that increased insulin levels in obesity can promote inflammation by directly reducing Treg suppression and thereby driving the chronic inflammation of obesity.

### Migration and activation of T cells in VAT in obesity

While the presence of inflammatory T cells in adipose tissue is now well established, the mechanism by which T cells are recruited into obese VAT and how they are activated has been less clear. Recent publications have started to address these questions. The chemokine receptor CXCR3 has recently been found to have a critical role in T cell recruitment into VAT in obesity. CXCR3 knockout mice on high-fat diet had fewer VAT T cells than wildtype mice on high-fat diet (55). Obese CXCR3 knockout mice also developed less insulin resistance following 8 weeks of high-fat diet compared to wildtype obese mice, although this protection was lost by 16 weeks. The diet-induced obese CXCR3 knockout mice also expressed lower levels of mRNA for several pro-inflammatory genes including MCP-1 in adipose tissue, and higher levels of anti-inflammatory genes, including Foxp3 and IL-10 (55). A separate group also examined CXCR3 knockouts on high-fat diet for 20 weeks. They observed similar weight gain in CXCR3-/- and wildtype mice, but observed decreased fasting glucose and improved glucose tolerance in CXCR3 knockouts, along with decreased infiltration of immature myeloid cells into VAT (56). Other chemokine systems may also play a role in this process. Expression of the chemoattractant RANTES is induced in adipocytes in obesity along with its chemokine receptor CCR5 (57, 58). CCR5 also has a critical role in obesity-associated inflammation as CCR5 knockout mice on high-fat diet demonstrate a shift from pro-inflammatory M1 to M2 macrophages in VAT, and are protected against insulin resistance in obesity (58).

Once T cells are recruited to adipose tissue in obesity, they require activation to maintain a pro-inflammatory state. There is evidence for both macrophages and B cells (51, 59), as well as adipocytes (60), in acting as antigen-presenting cells to stimulate T cell activation and inflammation of VAT in obesity. Adipocytes certainly secrete hormones and cytokines that are well known to promote inflammatory T cell activation, including leptin, resistin, TNF-α, and IL-6. Additionally, adipocyte-derived lipids have been shown to modulate T cell function (60). However, in addition to secreting cytokines, hormones, and lipids that can influence T cell inflammation, adipocytes may also serve as antigen-presenting cells by expressing MHC class II and co-stimulatory membrane receptors on their surface, thereby activating CD4+ T cells (61).

### Targeting T cell inflammation in the treatment of insulin resistance

Understanding the role of lymphocyte inflammation in promoting insulin resistance in obesity opens up new possibilities for treatment of type 2 diabetes. Weight loss has been shown to both decrease inflammatory cytokine production and improve insulin sensitivity (62). Even exercise alone has been shown to reduce inflammatory cells in VAT in obesity (63). However, as stated above, weight loss through lifestyle modifications of diet and exercise is challenging for many and oftentimes unsuccessful. In addition, new evidence supports the idea that weight loss may be harder to achieve in an inflammatory state. In a recent report published in The Journal of Clinical Endocrinology and Metabolism in 2014, the presence of increased inflammation, as measured by inflammatory cytokine levels, prior to bariatric surgery, led to decreased body mass index reduction following weight-loss surgery (64).

Subsequently, the idea of immunotherapy in the treatment of type 2 diabetes had gained considerable interest. Thus far, there have been a handful of targets of inflammation that have shown therapeutic promise for the treatment of diabetes. The first is TNF-α blockade, which has shown some mixed success in improving insulin resistance in diabetic patients (65–67). The second target of interest is NF-κB activation blockade. Early-animals studies in 2001 showed that salicylates could reverse obesity-associated insulin resistance in both genetically obese and diet-induced obese mice (68, 69), which resulted in decreased NF-κB activation. In a more recent series of studies, salicylates were found to be potentially useful for the treatment of high-fat diet-induced insulin resistance in diabetes in human subjects (70), yet results are mixed (71), and blockade of NF-κB is very downstream in the VAT inflammatory pathway in obesity, so inflammatory cell numbers are not typically affected (13). Finally, there is some evidence that selective blockade of IL-1 receptor activation may prevent insulin resistance in obesity-associated inflammation (72). A more recent study investigated the effect of T cell co-stimulation blockade on obesity-associated insulin resistance, and found that both CD40L antibody and cytotoxic T lymphocyte antigen 4 (CTLA-4) immunoglobulin (Ig)-treated diet-induced obese mice had reduced numbers of VAT macrophages and CD8+ T cells, as compared to obese mice treated with control antibody (73). However, only the CD40L antibody-treated, and not the CTLA-4 Ig-treated mice, had decreased weight gain and subsequent improvement in insulin sensitivity. Altogether, modulation of obesity-associated inflammation is of considerable interest at present, and may offer novel therapeutic targets for type 2 diabetes in obesity.

## Malnutriton and Immunodeficiency

Like obesity, malnutrition is a serious global health issue affecting many. In 2011, it was estimated that over 52 million children were severely malnourished (74). This has important health implications, as children that are malnourished have suppressed immunity and higher mortality due to infections (75–77). Childhood malnutrition is also associated with impaired cognitive development, as well as persistent defects in learning and memory (78, 79). Young children and infants are particularly at risk, as 45% of deaths in children under age five years are due to undernutrition (74). Malnutrition specifically accounts for 2.6 million childhood deaths annually due to infections including diarrheal illness and pneumonia (80, 81).

In addition to chronic malnutrition in developing countries, malnutrition can also affect immune function in other clinical settings in which nutritional status influences outcome. Such scenarios include patients with cancer- and AIDS-related cachexia, critically ill ICU patients, and low birth weight neonates with inadequate adipose stores. In fact, it is estimated that 60–80% of patients with advanced cancer are cachexic, and weight loss prior to chemotherapy greatly increases mortality in cancer patients (82, 83). Additionally, the U.S. National Cancer Institute has estimated that up to 40% of cancer deaths result from infections related to malnutrition. Malnutrition-induced immune suppression is, therefore, a major cause of morbidity and mortality in multiple susceptible patient populations. Despite an abundance of epidemiological evidence linking malnutrition and immunodeficiency, little is known about the impact of malnutrition on specific immune cell populations. Here, we describe recent findings on the role of malnutrition on lymphocyte number and function.

### Malnutrition and T cells

Malnutrition has been linked to immune dysfunction in a variety of settings, including starvation and cachexia in both human beings and mice (77, 80–82). As T cells are a vital component of the adaptive immune system, several studies have specifically examined the effect of malnutrition on T cell number and function. Mice fasted for 48 h had drastically decreased thymocyte and splenocyte counts compared to fed-control mice (84–86). Within the spleen, total T cell and CD4+ T cell numbers from fasted mice were decreased by 40–50% compared to control animals (85, 86). Additionally, mice fed a protein-deficient diet had atrophic spleens and decreased T cell numbers compared to control mice (87, 88). Decreased T cell numbers observed in fasted mice are mimicked in malnourished human beings. Malnourished children had decreased CD4+ and CD8+ T cell numbers in whole-blood samples compared to T cell numbers from well-nourished children (89).

In addition to drastically reducing T cell numbers, malnutrition also affects T cell cytokine production. Protein energy malnutrition impaired the ability of rat lymphocytes to proliferate and produce the Th1-associated cytokine IFN-γ (90). Similarly, T cells from mice that were fasted for 48 h and then activated *in vitro* had greatly decreased production of the Th1 cytokines IL-2 and IFN-γ, compared to T cells from fed control animals (86). This decrease in cytokine production observed in malnourished mice was also observed in human studies, as malnourished children had decreased levels of cytokines important for Th1 differentiation (IL-12, IL-18, and IL-21) as well as decreased Th1 cytokines IFN-γ and IL-2 (91). In a separate study, the same group showed that malnourished children had increased expression of Th2 cytokines IL-4 and IL-10 (92). Altogether, these findings suggest that malnutrition shifts the balance of pro-inflammatory Th1 versus anti-inflammatory Th2 cytokines, and may offer an explanation as to how malnutrition predisposes to infection.

Upon activation, effector T cells undergo metabolic reprogramming that results in transition from an oxidative state to a highly glycolytic phenotype. Accompanying this metabolic switch is an increase in expression of the ubiquitous glucose transporter, Glut1, which leads to increased glucose uptake and subsequent glycolysis (93, 94). This shift from oxidative to glycolytic metabolism is critical to maintain T cell function, as decreased glucose availability inhibits T cell cytokine production and proliferation (93, 95). We have found that acute malnutrition inhibits activation-induced T cell glucose metabolism (86). Rescue of this metabolic defect with T cell-specific overexpression of a Glut1 transgene normalizes glucose uptake to levels in T cells from fed controls. Additionally, Glut1 overexpression returns inflammatory cytokine production back to levels seen in T cells isolated and activated from fed mice, suggesting that rescue of T cell glucose metabolism reverses the T cell functional defects seen in malnutrition (86). This leads to the intriguing possibility that decreased circulating glucose levels in fasting may directly affect T cell metabolism and, therefore, T cell function. Indeed, anti-CD3 antibody treatment to activate T cells *in vivo* led to an increase in T cell glucose uptake and a marked decrease in circulating glucose levels, resulting in hypoglycemia in mice (96). Hypoglycemia following anti-CD3 antibody treatment did not occur in Rag1-/- mice that lack lymphocytes (96). This suggests that T cells utilize a substantial amount of glucose during activation and may be affected by altered circulating glucose levels due to starvation or malnutrition. More work is needed to gain a complete understanding of the relationship between malnutrition, T cell metabolism, and function.

### Malnutrition and infection

Increasing evidence suggests that malnutrition can lead to more severe viral infections and can affect vaccine responses in children (76, 97). Although most epidemiological studies examined general nutrient deficiencies, many of the animal studies specifically assess the effect of protein energy malnutrition (PEM) on viral immunity, using low-protein or protein-deficient diets in mouse models (87, 88, 98, 99). In a respiratory infection model, mice fed a low-protein diet required a 1000-fold lower viral titer for 50% lethality than normal fed controls (87). Similarly, malnourished mice inoculated with *Mycobacterium tuberculosis* had 2–3 logs more bacilli in the lungs than mice receiving a full protein diet (100). PEM also increased the susceptibility to influenza infection due to impaired viral clearance and decreased lymphocyte numbers (88). Importantly, supplementation with protein enhanced viral clearance and decreased mortality from influenza (88).

Furthermore, PEM impaired the homeostatic proliferation of mouse memory CD8+ T cells in response to lymphocytic choriomeningitis virus (LCMV) (98). A separate group showed a decreased number of viral-specific CD8+ T cells upon protein malnutrition during LCMV infection (99). The low-protein diet impaired the recall response and maintenance of memory CD8+ T cells; this could be rescued by protein supplementation, suggesting that dietary protein is critical for maintaining a functional pool of memory T cells (98). This defect in CD8+ T cell function during malnutrition also occurs in human beings. Malnourished children hospitalized with bacterial infections were shown to have a lower fraction of memory T cells than well-nourished infected controls (101), as well as decreased total CD4+ and CD8+ T cell numbers (89). Together, these data suggest that malnutrition decreases CD8+ T cell number and function and predisposes to infection.

### Malnutrition and autoimmunity

As malnutrition reduces T cell number and function, there is increasing evidence that calorie-restriction and fasting protect against autoimmune disease. One study examined BXSB mice, which spontaneously develops an autoimmune disease similar to systemic lupus erythematosus (SLE) (102). A 40% calorie-restriction inhibited the onset of autoimmunity and increased the lifespan of mice compared to those on a non-restricted diet (103). The calorie-restricted mice also had decreased IL-2 production and lymphocyte proliferation compared to controls (103). Piccio et al. examined a 40% calorie-restriction in the context of two EAE models. In both EAE models, calorie-restriction increased survival and decreased disease scores during EAE progression (104). Additionally, calorie-restriction significantly decreased central nervous system inflammation and demyelination as well as plasma levels of the pro-inflammatory cytokine IL-6 (104). A separate group showed that mice fasted for 48 h had decreased EAE disease scores as well as decreased IFN-γ production (105). Similar findings were also shown in calorie-restricted rats (106). Interestingly, mice fed *ad libitum* every other day (intermittent feeding) also developed less severe EAE compared to control animals fed *ad libitum* (107). Together, these data suggest that calorie-restriction and fasting prevent autoimmunity, likely by decreasing T cell responses and inflammatory cytokine production.

## Role of Leptin in Mediating Nutritional Effects on T Cells

Leptin is an adipokine secreted in proportion to adipocyte mass. In addition to its well-described role in regulating appetite, energy expenditure, and body weight, leptin is also a pro-inflammatory cytokine. Leptin has direct and indirect effects on T cell number and function, promoting Th1 and Th17 cell number and cytokine production while inhibiting Th2 cytokine production and Treg proliferation (108). The effects of leptin on T cell number and function have been extensively reviewed and will not be discussed in depth here, yet special consideration must be given to the role of leptin in mediating T cell changes in malnutrition or obesity.

Acute starvation causes a drastic reduction in leptin levels in both mice and human beings; indeed, many studies utilize fasting as a way to model hypoleptinemia in mice (84–86). Low levels of leptin are associated with high rates of death from infectious diseases (109, 110). In a recent study published in the Journal of Clinical Endocrinology and Metabolism, the hormonal and metabolic status of malnourished Ugandan children was examined. In this study, low leptin levels were found to be the single most important biomarker to predict mortality during inpatient treatment of malnutrition (111).

Additionally, leptin-deficient *ob*-/- mice, despite their genetic obesity, have many features similar to those seen in malnutrition, including decreased body temperature, infertility, and low metabolic rate (112). *Ob*-/- mice also have immune defects similar to those seen in malnourished human beings and animals, including decreased thymocyte and splenocyte numbers, increased susceptibility to infection, and protection against certain forms of autoimmunity (84). Both the metabolic and immune defects seen in *ob*-/- mice can be reversed by treatment with recombinant leptin.

**Figure 2:**
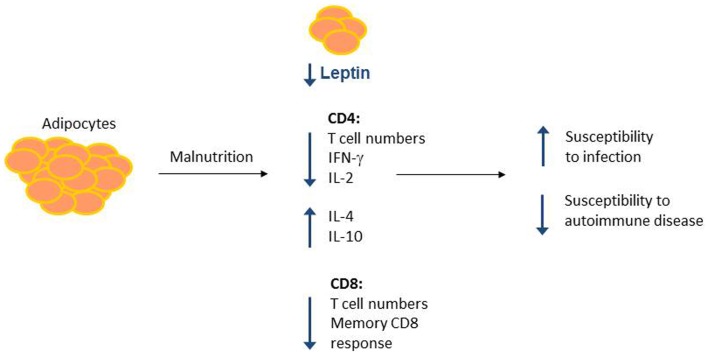
**Malnutrition leads to changes in adipocyte-secreted hormones and T cell number and function**. Malnutrition leads to decreased adipocyte mass, which results in decreased circulating leptin. Concurrently, malnourished individuals show changes in CD4+ and CD8+ T cell number and function, leading to increased susceptibility to infection and protection against certain forms of autoimmunity. Leptin may mediate a subset of these malnutrition-related changes to T cell number and function, as leptin is known to alter CD4+ T cell number and cytokine production.

A role for leptin in reversing malnutrition-induced immunosuppression has been examined by our group and others. Although fasting leads to decreased T cell number and function, these defects can be reversed by treatment with recombinant leptin during the period of starvation (85, 86). Either leptin administration to fasting mice *in vivo* or leptin treatment of T cells isolated from fasted animals *in vitro* was sufficient to rescue inflammatory cytokine production in activated T cells from fasted mice (86). Importantly, leptin also rescued the T cell metabolic defects seen in fasting. Leptin treatment led to increased Glut1 expression and increased glucose uptake and glycolysis in fasted animals (86). Leptin is, therefore, a critical regulator of T cell glucose metabolism to fuel T cell activation. In other studies, leptin replacement was found to reverse starvation-induced immunosuppression, as measured by a delayed-type hypersensitivity (DTH) response (113), and prevented starvation-induced protection against EAE (105). Together, these data suggest that leptin is an important modulator of nutritional effects on T cell metabolism and function (Figure 2).

Leptin itself or the regulatory pathways that control leptin expression or action may be important new targets to promote immunity (114). In a small clinical study of malnourished children hospitalized for infection in Mexico, treatment of peripheral blood T cells with leptin *in vitro* increased T cell activation and inflammatory IL-2 and IFN-γ cytokine production, while decreasing IL-4 and IL-10 production (115). Additionally, leptin has been used as a mucosal vaccine adjuvant for *Rhodococcus equi* bacterial infections in mice, and leptin signaling was also associated with higher *Helicobacter pylori* antibody titers following vaccination (116, 117). Understanding the pathways by which leptin regulates T cell metabolism and function may lead to new ways to augment immunity in select clinical disorders associated with undernutrition.

Conversely, the fact that leptin is a pro-inflammatory cytokine secreted by adipocytes, in proportion to adipocyte mass, points to a role for leptin in obesity-associated inflammation and insulin resistance, and many have speculated on the role for leptin in promoting inflammation in obesity. This is complicated, however, by the fact that both the leptin-deficient and leptin receptor-deficient *ob*-/- and *db*-/- mice, respectively, still develop insulin resistance in the setting of genetic obesity (118). As leptin acts on every cell in the immune system as well as stromal cells that influence immune cell development, the direct effect of leptin on lymphocyte and macrophage inflammation in obesity remains unclear.

## Nutrient-Sensing T Cell Signaling

Key molecular signals are involved in T cell metabolic adaptation during nutrient stress; among them, the metabolic regulator AMP-activated protein kinase (AMPK) and its upstream kinase, liver kinase B1 (LKB1), have critical roles in regulating T cell number, function, and metabolism (119). AMPK is a well-described metabolic regulator that responds to energy stress and depletion of ATP reserves. AMPK is maximally active when bound to AMP and phosphorylated by an upstream kinase, typically LKB1. In select tissues, AMPK may also be phosphorylated by alternative kinases, including calcium/calmodulin-dependent protein kinase kinase 2 (CaMKK2), which is responsive to changes in intracellular Ca2+ concentration (120). In fact, in T cells, activation of AMPK via phosphorylation by CaMKK2 occurs following TCR activation, presumably in anticipation of the increased energy demands of activation (120). AMPK activation leads to increased energy-producing/catabolic pathways while inhibiting energy-consuming/anabolic pathways. Interestingly, the diabetic drug metformin activates AMPK, which leads to improved insulin sensitivity and increased glucose uptake in metabolic cells.

We and others have found that LKB1 is a central regulator of T cell number, activation, and metabolism (121–123). T cell knockout of LKB1 resulted in blocked thymocyte development and decreased numbers of both CD4+ and CD8+ T cells. LKB1-knockout T cells had defects in both viability and proliferation, as well as increased expression of inflammatory cytokines IFN-γ and IL-17 and increased glucose metabolism (121). AMPK-knockout reproduced only a portion of the defects seen in the LKB1-knockout mouse. Loss of T cell AMPK did not alter thymocyte development or T cell number, but did result in increased expression of inflammatory cytokines as well as increased glucose metabolism. Increased mTOR complex 1 (mTORC1) signaling in both AMPK- and LKB1-knockouts, contributed to the phenotype (121).

The nutrient-sensing mTOR pathway is also important for cellular nutrient/energy sensing and drives inflammatory T cell differentiation and function. T cell specific knockout of mTOR kinase to delete both mTORC1 and mTORC2 suppresses effector T cell generation but allows Treg differentiation (124). The specific contribution of mTORC1 activity to Treg function appears to be complex. Deletion of mTORC1 activity has been shown to prevent Treg suppressive function in some settings, and not affect Treg in others (125, 126). AMPK has also been shown to promote Treg cell function while inhibiting signaling pathways, including mTORC1, which promote effector T cell differentiation, particularly Th1 and Th17 (127). The role of the nutrient-sensing mTOR and AMPK pathways on T cells in malnutrition, therefore, may be to act as metabolic checkpoints so that inflammatory T cell expansion is limited when nutrient availability is low. In the context of obesity, adipokines such as leptin may promote mTOR activity to promote effector T cell generation and contribute to inflammation (85).

## Conclusion

**Figure 3:**
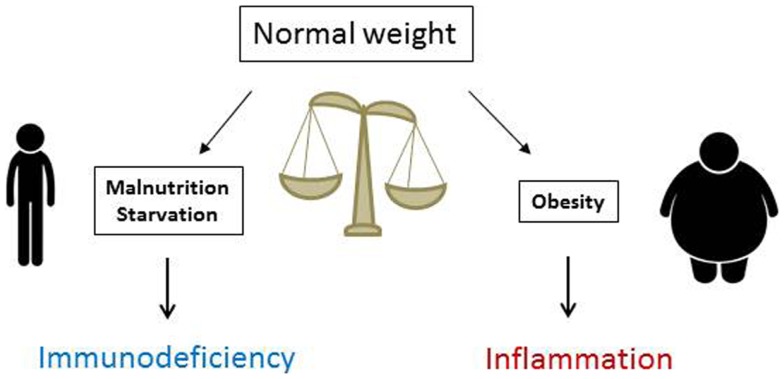
**Nutritional status is critical for normal immune function**. Malnutrition reduces immunity and increases markedly the risks and mortality from severe infections, whereas obesity heightens immune reactivity and predisposes to systemic inflammation that fosters the development of inflammatory disorders and insulin resistance leading to type 2 diabetes mellitus.

In summary, T cell number, function, and metabolism are significantly affected by nutritional status. Whereas malnutrition lowers T cell number and metabolism and increases susceptibility to infection, obesity increases inflammatory T cell numbers in VAT and promotes systemic inflammation (Figure 3). Understanding how T cells are altered in obesity and malnutrition will lead to better understanding of and treatment for diseases where nutritional status determines clinical outcome. Targeting the inflammatory T cell response in obesity offers novel therapeutic options for insulin resistance and type 2 diabetes, whereas augmenting T cell response in malnutrition may promote immunity in clinical scenarios where malnutrition leads to poor outcome. Such scenarios could include augmenting vaccine response in malnourished children from underdeveloped countries and protecting chronically ill patients with cachexia against infection. An understanding of nutritional effects on T cells is therefore critically important for public and global health.

## Conflict of Interest Statement

The authors declare that the research was conducted in the absence of any commercial or financial relationships that could be construed as a potential conflict of interest.
